# Retention of fellows to the faculty at a research-intensive academic medical center: a mixed-methods study

**DOI:** 10.1080/10872981.2026.2714642

**Published:** 2026-08-07

**Authors:** Janice L. Hanson, Maria Pérez, Yaheng Lu, Eva M. Aagaard, Donna B. Jeffe

**Affiliations:** a Washington University School of Medicine, St. Louis, Missouri, USA

**Keywords:** Medical education, graduate medical education, fellowship training, faculty recruitment, mixed methods

## Abstract

**Background:**

As clinical practice provides >50% of medical schools’ revenue, research-intensive medical schools often seek to retain fellows with clinical-care interests. Fellows’ considerations when deciding whether to stay at research-intensive institutions where they trained remain understudied. We sought to identify experiential factors associated with faculty-position offers and decisions of fellows in training.

**Methods:**

We conducted a mixed-methods study using a survey and semi-structured interview. We invited 471 fellows training in all clinical departments at our institution to complete an anonymous survey between April and June 2021 to examine their level of agreement (strongly disagree [1] to strongly agree [7]) with positive training and mentoring experiences and their academic-medicine career goals in association with being offered a position and, if offered, accepting or declining. After survey completion, we invited fellows for interviews about their decision-making to seek, accept, or decline post-fellowship faculty-positions as well as what our institution could do to encourage fellows to accept offered positions.

**Results:**

We analyzed survey data from 168 (35.7%) fellows. Of 48 fellows who were offered faculty positions, 21 accepted and 27 declined; 56 fellows were not offered a position and 64 were too early in training to have received an offer. Fellows who were offered/accepted positions reported higher mean (SD) agreement with positive training (5.9 [1.2]; *p* < 0.02) and mentoring (6.1 [1.0]; *p* < 0.001) experiences and having academic-medicine career goals (5.8 [0.8]; *p* < 0.001) than fellows in other faculty-offer groups. In interviews, 24 fellows reported career goals and institutional resources as influencing decisions. Fellows described institutional opportunities for intervention, especially enhanced communication about institutional priorities and potential positions.

**Conclusions:**

Clear communication with clinical fellows about their academic-medicine career goals and fellowship-training and mentoring experiences can inform decision-making about faculty offers and better equip institutional leaders to retain fellows who align with institutional needs.

## Background

Academic physicians educate and mentor medical students, residents, and fellows, conduct cutting-edge research, and provide patient care to fulfil the tripartite mission of academic medicine [1]. In 2021, 42% of U.S. medical graduates completing the Association of American Medical College’s (AAMC) annual Graduation Questionnaire reported planning to pursue academic-medicine faculty positions during their careers [2]. Twenty percent of physicians who completed residency training between 2011 and 2020 held a full-time faculty position at U.S. medical schools in 2021 [3]. As clinical practice provides more than half of medical schools’ revenue [4], and they face needs for increased clinical-enterprise growth to support the research and education missions [1], research-intensive institutions may seek to retain physicians who primarily provide clinical care, a group not traditionally recruited in research-intensive institutions.

National-cohort studies have identified several variables positively associated with academic-medicine faculty appointment including conducting research and/or authorship on publications during medical school and residency, graduating with MD/PhD (vs. MD or MD/other-advanced) degrees [5–7], and among MD/PhD graduates attending Medical Scientist Training Programme (MSTP)-funded medical schools [8]. Medical school-related debt has been associated with a lower likelihood of choosing an academic-medicine career among anaesthesiology interns, residents, and fellows [9], and positive-mentoring experiences during fellowship have been associated with pursuing an academic-medicine career [10]. However, fellows’ decision-making about whether to stay at the institution where they trained remains understudied. With 28,868 fellows training in subspecialties in the U.S. in 2023-2024 [11], fellows can be an important source of future faculty. Graduating residents may apply to fellowship positions using the Electronic Residency Application Service® (ERAS®) [12], wherein fellows apply to their desired subspecialty. Applicants are evaluated by fellowship programmes through a combination of application review and interviews. Following interviews, applicants rank their preferred programmes and programmes rank their preferred applicants in ERAS. An algorithmic process matches applicants to a fellowship programme based on these ranked preferences. In smaller subspecialty fellowships, most commonly surgical and non-clinical (often research or academic career pathway) fellowships, the application process may occur outside of ERAS through direct application and interview. Fellows earn wages as employees of the institution where they train. Fellowships may include either or both subspecialty training after residency and focused preparation for an academic career, but fellowships are not degree programmes. Understanding fellows’ decision-making might assist fellowship programme directors in recruiting and guiding fellows’ career development during training to align with and support fellows’ career goals [13] as well as gauge and promote a fellow’s interest in staying at the institution where they trained to meet institutional and departmental needs, if a position is available.

To investigate fellows’ decisions to remain at their institution of training, we planned a mixed-methods study at Washington University School of Medicine (WashU Medicine) to identify variables associated with fellows’ decisions to join our faculty, to explore reasons for their decisions, and to examine what can be done to encourage fellows to accept offered positions.

## Methods

### Study design and participants

This study was conducted at an urban medical school associated with an R1 university in a midwestern U.S. city. The R1 Carnegie Classification of Institutions of Higher Education indicates a doctoral-granting university with very high research activity [14]. Based on total year-end composite data for fiscal year 2025 [15], our research-intensive medical school is highly ranked in the Top 10 for direct, U.S. federal expenditures for research, grants, and contracts. Using a sequential triangulation design [16], we first administered a 35-item anonymous Qualtrics (Qualtrics, Provo, Utah) survey to fellows between April and June 2021 to identify variables associated with career plans and decisions to join or not join faculty after fellowship (supplementary material). At the survey’s end, fellows were invited to participate in an interview about their training experiences and career plans (supplementary material). We triangulated our survey and interview findings.

### Step 1: survey

Twenty-one items used a 7-point Likert scale from (1) strongly disagree to (7) strongly agree, including 11 items previously associated with plans to pursue academic-medicine careers [17] or mentors’ roles [18–20]; 10 items were newly-created about post-fellowship career decisions. Of the 21 survey items, sixteen items inquired about fellows’ training experiences and academic-medicine career goals; among fellows who reported having a mentor, five items inquired about mentoring experiences. Demographics were obtained (gender, race, Hispanic ethnicity, marital status, year in training, terminal degrees, fellowship department, total current debt, and influence of debt on career plans) and participants could select ‘prefer not to answer’ in response to items about gender, race, Hispanic ethnicity, and total current debt. A 7-category variable measured fellow’s career-activities intentions: full-time university faculty position in 1) basic science teaching/research, 2) clinical teaching/research, 3) clinical focus, including quality and safety or clinical practice/leadership; 4) non-academic clinical practice; 5) non-academic research-scientist position; 6) any other career; or 7) undecided. We asked whether fellows considered joining our faculty (yes/no), whether fellows had been offered a faculty position, and, among fellows offered a position, if they accepted the offer.

We used an iterative process of principal components analysis (PCA) with varimax rotation for data reduction and to examine the underlying component structure of 16 items measuring fellows’ agreement with statements about positive-training experiences and academic-medicine career goals. At each step, we retained only items with moderate-to-high component saturation (i.e., loadings ≥ .60) on a single component, and we eliminated items with weak loadings < .40 all components, indicating that the items are not measuring the variable(s) identified by one or more emerging components; we also dropped items loading ≥ .40 on more than one component to ensure that each item is measuring an aspect of only one component. These decision rules to retain items with moderate-to-high loadings help ensure the stability of findings across studies [21,22], as a sample’s component pattern with substantial component saturation (i.e., loadings ≥ .60) can approximate a good fit to the population pattern across different conditions when sample sizes include at least 150 participants [21]. A separate PCA was run with the five mentoring-experience items, because only fellows who indicated they had a mentor could respond to these items. Cronbach’s alpha measured the internal-consistency reliability of items with moderate-to-high loadings on one component. Mean scores of items loading on each component were computed; higher scores indicated stronger agreement with positive training and mentoring experiences and endorsement of academic-medicine career goals.

We used chi-square tests of associations among categorical variables and reported frequencies (%) of fellows in each category. One-way analysis of variance of fellows’ training and mentoring experiences and endorsement of academic-medicine career goals, with Bonferroni post-hoc pairwise comparisons, tested between-groups differences in each continuous measure by fellows’ demographics, career-activity intentions, consideration of joining our faculty, and faculty-offer groups (outcome of interest); we report the mean [SD] of scores in each category. Two-tailed *p* < 0.05 was considered statistically significant. IBM SPSS version 28.0 (IBM Corp., Armonk, New York) was used for statistical analyses.

### Step 2: interview

The semi-structured interview explored fellows’ perspectives, experiences, and interpretations of their conversations with leadership about job opportunities, their reasons to seek, accept, or decline a faculty position, suggestions for what could be done to encourage fellows to accept positions, and the value placed on faculty roles at our institution. Open-ended interview questions were developed to prompt discussion. Since the survey data were collected anonymously, participants’ survey and interview data could not be linked to individual fellows who participated in both. The co-first author (MP), a project manager with extensive training and experience in conducting interviews for medical education research studies, completed all interviews via Zoom. Working in the Office of Education’s Medical Education Research and Evaluation Unit primarily in studies of medical students, her project-manager position at the medical school did not involve routine interactions with fellows. Without a position of authority in the organisational hierarchy and with substantial experience conducting research interviews, the interviewer tried to create an open and respectful atmosphere, allowing fellows to answer interview questions honestly, thus minimising the potential for social-desirability response bias due to a power imbalance, which might be expected to occur if the interviewer had been on the faculty or in another position of power. Audio recordings were transcribed verbatim.

None of the study team members who interviewed and analysed interview data supervised fellows. The three team members who conducted the qualitative analysis do not teach, mentor, or work with fellows in patient care settings; their positionality relative to the fellows was that of researchers interested in learning about fellows’ experiences and perspectives. We used inductive thematic analysis to identify codes, themes, and relationships between codes and themes [23]. Consistent with a philosophical framework of social constructivism [24], this approach recognised individual differences of experience and interpretation while seeking commonalities of perspectives that defined themes [25]. All transcripts were coded independently by at least two of three authors (JH, MP, YL) and reviewed by a third team member. Once we had a common codebook, pairs of reviewers coded the remaining transcripts; each pair met to address coding differences. Frequent team discussions to reconcile coding differences and refine the codebook supported reflexivity during data analysis and consistency in interpretation. This helped ensure trustworthiness as we identified and set aside pre-existing beliefs and preconceptions and engaged in self-reflection to focus on fellows’ perceptions [26]. We confirmed we had reached data sufficiency when we identified no new codes in later transcripts. Codes were grouped conceptually; each group of codes defined a theme.

### Step 3: mixed-methods analysis and synthesis

Using a sequential triangulation mixed-methods design, we first completed quantitative analysis of the survey data and then thematic analysis of the interview data; next we examined both sets of results to identify how the survey data corresponded to the interview themes. The research team triangulated the survey and interview results, adding depth and detail to the survey findings. The team also discussed how fellows’ perspectives in the interviews enriched our understanding of and provided additional insights into fellows’ decision-making process [16]. This mixed-methods study was reviewed by the Institutional Review Board (IRB) at WashU Medicine and was approved as exempt research (#202103193). All participants received written information about the study (including the study team’s contact information should a participant need to ask questions) and provided implied consent via voluntary participation in the survey and interview following review of the study-information sheet, consistent with our IRB’s guidance for minimal-risk and exempt human-subjects research. This study was performed in accordance with the principles stated in the Declaration of Helsinki.

## Results

### Survey

Of 467 physician fellows invited to participate across 15 clinical departments, 168 (36.0%) completed the survey and were included in the sample. Table 1 shows the sample’s characteristics. Forty-eight fellows were offered a faculty position (21 accepted, 27 declined); 56 fellows were not offered a position, and 64 were too early in training to have received an offer. This four-category faculty-offer variable did not differ significantly by gender, race/ethnicity, or other demographic variables. Most respondents (134/168 [79.8%]) considered joining the faculty at our institution, including 46 of 48 (95.8%) fellows offered positions and 55 of 64 (85.9%) fellows too early in training to have received an offer (*p* *<* 0.001).

**Table 1.** Frequencies (%) of demographic and other characteristics of fellows surveyed, by faculty-offer group.

Characteristics	Total^a^ *N* = 168 (%)	Offered/ accepted position^b^ *n* = 21 (12.5%)	Offered/ declined position^b^ *n* = 27 (16.1%)	Not offered a position^b^ *n* = 56 (33.3%)	Too early in training to receive an offer^b^ *n* = 64 (38.1%)	*p* *^c^*
Gender						0.92
Male	89 (53.0)	11 (12.4)	15 (16.9)	30 (33.7)	33 (37.1)	
Female	74 (44.0)	10 (13.5)	12 (16.2)	23 (31.1)	29 (39.2)	
Non-binary/Prefer not to answer	5 (3.0)	0 (0.0)	0 (0.0)	3 (60.0)	2 (40.0)	
Race/Ethnicity						
White	110 (65.5)	12 (10.9)	21 (19.1)	35 (31.8)	42 (38.2)	0.91
Asian	31 (18.5)	6 (19.4)	3 (9.7)	10 (32.3)	12 (38.7)	
URiM	16 (9.5)	2 (12.5)	2 (12.5)	6 (37.5)	6 (37.5)	
More than one race or Unknown	11 (6.5)	1 (9.1)	1 (9.1)	5 (45.5)	4 (36.4)	
Marital Status						0.64
Married/partnered	124 (73.8)	18 (14.5)	19 (15.3)	40 (32.3)	47 (37.9)	
Divorced/separated/never married	39 (23.2)	3 (7.7)	6 (15.4)	14 (35.9)	16 (41.0)	
Prefer not to answer	5 (3.0)	0 (0.0)	2 (40.0)	2 (40.0)	1 (20.0)	
Year of fellowship						0.34
Year 1	89 (53.0)	8 (9.0)	11 (12.4)	32 (36.0)	38 (42.7)	
Year 2	43 (25.6)	5 (11.6)	7 (16.3)	13 (30.2)	18 (41.9)	
Year 3	26 (15.5)	6 (23.1)	7 (26.9)	8 (30.8)	5 (19.2)	
Year 4 or more	10 (6.0)	2 (20.0)	2 (20.0)	3 (30.0)	3 (30.0)	
Degree						0.33
MD	142 (84.5)	19 (13.4)	24 (16.9)	48 (33.8)	51 (35.9)	
DO or MBBS	13 (7.7)	0 (0.0)	3 (23.1)	5 (38.5)	5 (38.5)	
MD/PhD	13 (7.7)	2 (15.4)	0 (0.0)	3 (23.1)	8 (61.5)	
Total debt (quartiles)						0.81
No debt	49 (29.2)	6 (12.2)	1 (2.0)	21 (42.9)	21 (42.9)	
$50,000-$99,999	26 (15.5)	4 (15.4)	2 (7.7)	10 (38.5)	10 (38.5)	
$100,00-$249,999	36 (21.4)	5 (13.9)	9 (25.0)	12 (33.3)	10 (27.8)	
$250,000-$300,000	54 (32.1)	6 (11.1)	14 (51.1)	13 (24.1)	21 (38.9)	
Prefer not to answer	3 (1.8)	0 (0.0)	1 (33.3)	0 (0.0)	2 (66.7)	
Influence of debt on career plans						0.22
No influence	73 (43.5)	12 (16.4)	7 (9.6)	28 (38.4)	26 (35.6)	
Minor influence	48 (28.6)	6 (12.5)	7 (14.6)	14 (29.2)	21 (43.8)	
Moderate influence	30 (17.9)	3 (10.0)	9 (30.0)	7 (23.3)	11 (36.7)	
Strong influence	17 (10.1)	0 (0.0)	4 (23.5)	7 (41.2)	6 (35.3)	
Considered staying after fellowship						<0.001
Yes	134 (79.8)	21 (15.7)	25 (18.7)	33 (24.6)	55 (41.0)	
No	34 (20.2)	0 (0.0)	2 (5.9)	23 (67.6)	9 (26.5)	
Career-activities intentions^d^						<0.001
Faculty: Basic science teaching/research	10 (6.0)	2 (20.0)	0 (0.0)	3 (30.0)	5 (50.0)	
Faculty: Clinical teaching/research	55 (32.7)	12 (21.8)	7 (12.7)	9 (16.4)	27 (49.1)	
Faculty: Clinical focus	47 (28.0)	7 (14.9)	7 (14.9)	14 (29.8)	19 (40.4)	
Non-academic clinical practice	38 (22.6)	0 (0.0)	11 (28.9)	20 (52.6)	7 (18.4)	
Other/Undecided	18 (10.7)	0 (0.0)	2 (11.1)	10 (55.6)	6 (33.3)	

Abbreviations: URiM, underrepresented in medicine, (Black/African American or Hispanic/Latino[a]); DO, Doctor of Osteopathic Medicine; MBBS, Bachelor of Medicine, Bachelor of Surgery.

^a^ Percentages shown in column 2 are of the column total, N = 168.

^b^ Percentages in parentheses in columns 3–6 are the percentages of each row total shown in column 2.

^c^ Tests of significance were Chi-squared tests.

^d^ No fellows reported non-academic research-scientist career-activities intentions.

We observed a significant association between career-activities intentions and the proportions of fellows in the four faculty-offer groups (*p* *<* 0.001). All 21 fellows who accepted offers reported faculty-career-activities intentions; among the 27 fellows who declined positions, 14 reported faculty-career-activities intentions in clinical teaching/research or with a clinical focus (quality/safety or clinical practice/leadership); another 13 reported non-academic clinical practice or other/undecided career-activities intentions. Among the 64 fellows too early in training, 51 selected faculty-career-activities intentions (46 with clinical teaching/research and clinical-focus intentions).

Table 2 shows a 12-item, 2-component solution from the PCA of the 16 items about fellows’ training experiences and academic-medicine career goals. Four items were sequentially dropped from further analysis after four iterations of the PCA as these items were not descriptive of either of the two new variables (i.e., components) that emerged from the PCA. Items with moderate-to-high loadings on each component demonstrated high internal-consistency reliability: a 9-item component for fellows’ positive-training experiences (Cronbach’s alpha = 0.94) and 3-item component endorsing academic-medicine career goals (Cronbach’s alpha = 0.75). Among 168 fellows, 143 (85.1%) reported having a mentor and responded to mentor-role items; a separate PCA with these items yielded a single-component solution assessing positive-mentoring experiences (Table 3; Cronbach’s alpha = 0.84).

**Table 2.** Results of principal component analysis with varimax rotation (*N* = 168)^a^.

	Positive-training experiences Cronbach’s alpha = 0.94	Academic-medicine career goals Cronbach’s alpha = 0.75	
My professional values are well-aligned with [this institution’s] mission.	**0.58**	0.28	
I am satisfied with the role my training programme has had in my career advancement.	**0.82**	0.08	
[This institution] provides a collegial and inclusive work environment for me.	**0.85**	0.08	
[This institution] provides a supportive work environment for me.	**0.87**	0.06	
[This institution] is committed to my career success.	**0.90**	0.10	
[This institution] facilitates my professional development.	**0.90**	0.11	
I am satisfied with the mentoring I have received during fellowship.	**0.84**	0.14	
I am satisfied with the career advice I have received during fellowship.	**0.82**	0.25	
My fellowship training has provided me with opportunities to gain expertise in non-clinical career areas of interest to me.	**0.68**	0.33	
I am interested in pursuing a career in academic medicine.	0.26	**0.87**	
Protected time to conduct research is an important consideration as I plan my professional career path.	0.20	**0.73**	
Participation in education of others is an important consideration as I plan my professional career path.	−0.04	**0.82**	

^a^ Coefficients in bold font denote moderate-to-high component loadings on each component.

**Table 3.** Results of principal component analysis of five items measuring mentoring experiences among fellows who reported having a mentor (*N* = 143)^a^ ^,^ ^b^.

	Positive mentoring experiences Cronbach’s Alpha = 0.84
My mentor thinks highly of me.	0.726
My mentor guides my professional development.	0.888
My mentor suggests strategies for achieving career aspirations.	0.915
My mentor brings my accomplishments to the attention of important people.	0.842
My mentor has encouraged me to pursue a position here at [our institution].	0.614

^a^ Only one component was extracted; the solution cannot be rotated.

^b^ The first four items were previously published [18,19].

We observed statistically significant main effects of faculty-offer group on fellows’ agreement with positive-training experiences, academic-medicine career goals, and positive-mentoring experiences (Table 4). Post-hoc tests (each *p* < 0.05) indicated that fellows who accepted faculty positions reported higher mean agreement with academic-medicine career goals than fellows who declined or were not offered a position. Post-hoc comparisons (each *p* < 0.03) also indicated that fellows not offered a position reported lower agreement with positive-training experiences and positive-mentoring experiences than fellows too early in training; fellows not offered a position reported lower agreement with positive-mentoring experiences than fellows who accepted or declined a faculty offer (each *p* < 0.03). Fellows who considered staying at our institution reported significantly greater agreement with positive-training experiences, academic-medicine career goals, and positive-mentoring experiences than fellows who did not consider staying (each *p* < 0.03). Fellows graduating with an MD/PhD also reported greater agreement with positive-training experiences, academic-medicine career goals, and positive-mentoring experiences than fellows graduating with MD degrees (each *p* < 0.03).

**Table 4.** One-way analyses of variance of the newly developed multi-item scales from principal components analysis, by sample characteristics.

Characteristics	Positive-training experiences^a^ Mean (SD) *N* = 168	*p*	Academic-medicine career goals^b^ Mean (SD) *N* = 168	*p*	Positive-mentoring experiences^c^ Mean (SD) *N* = 143	*p*
Gender		0.56		0.17		0.46
Male	5.6 (1.2)		5.4 (1.4)		5.7 (1.1)	
Female	5.7 (1.1)		5.3 (1.2)		5.7 (1.1)	
Non-binary/Prefer not to answer	5.1 (1.4)		4.2 (2.0)		5.0 (1.6)	
Race/ethnicity		0.68		0.53		0.06
White	5.6 (1.2)		5.4 (1.3)		5.7 (1.0)	
Asian	5.5 (1.4)		5.4 (1.3)		5.7 (1.3)	
URiM	5.9 (0.8)		4.9 (1.6)		6.1 (0.7)	
More than one race/Unknown	5.5 (1.1)		5.2 (1.5)		4.8 (1.5)	
Marital status		0.52		0.88		0.26
Married/partnered	5.7 (1.1)		5.3 (1.4)		5.8 (1.0)	
Divorced/separated/never married	5.6 (1.3)		5.4 (1.2)		5.4 (1.3)	
Prefer not to answer	5.1 (0.9)		5.1 (2.0)		5.6 (0.7)	
Year of fellowship		0.32		0.09		0.34
Year 1	5.6 (1.1)		5.1 (1.5)		5.5 (1.2)	
Year 2	5.8 (1.1)		5.7 (0.8)		5.9 (0.9)	
Year 3	5.4 (1.5)		5.4 (1.2)		5.9 (0.9)	
Year 4 or more	6.1 (0.7)		5.9 (1.5)		5.7 (0.8)	
Degree		0.02		0.005		0.006
MD	5.6 (1.2)		5.2 (1.4)		5.6 (1.1)	
DO or MBBS	5.4 (1.2)		5.4 (1.4)		5.7 (0.9)	
MD/PhD	6.5 (0.6)		6.5 (0.5)		6.6 (0.5)	
Total debt (quartiles)		0.90		0.70		0.49
No debt	5.8 (1.3)		5.5 (1.6)		5.9 (1.2)	
$50,000-$99,999	5.5 (1.1)		5.3 (0.9)		5.5 (1.1)	
$100,00-$249,999	5.7 (1.0)		5.4 (1.0)		5.4 (1.2)	
$250,000-$300,000	5.6 (1.2)		5.2 (1.5)		5.7 (0.9)	
Prefer not to answer	5.5 (1.1)		4.7 (2.0)		6.2 (0.0)^d^	
Influence of debt on career plans		0.26		0.07		0.17
No influence	5.7 (1.3)		5.5 (1.3)		5.8 (1.2)	
Minor influence	5.8 (1.1)		5.4 (1.3)		5.6 (1.1)	
Moderate influence	5.7 (0.8)		5.3 (1.2)		5.8 (0.6)	
Strong influence	5.1 (1.4)		4.5 (1.6)		5.0 (1.5)	
Faculty-offer group		0.02		<0.001		<0.001
Offered/accepted position	5.9 (1.2)		5.8 (0.8)		6.1 (1.0)	
Offered/declined position	5.5 (1.0)		4.8 (1.2)		5.9 (0.8)	
Not offered a faculty position	5.3 (1.3)		4.9 (1.6)		5.1 (1.2)	
Too early in training to receive an offer	5.9 (1.0)		5.8 (1.1)		5.9 (0.9)	
Considered staying at our institution		0.02		<0.001		0.008
Yes	5.7 (1.1)		5.6 (1.1)		5.8 (1.0)	
No	5.2 (1.3)		4.4 (1.8)		5.1 (1.4)	
Career-activities intentions		<0.001		<0.001		<0.001
Faculty: basic science teaching/research	6.6 (0.5)		6.5 (0.3)		6.5 (0.9)	
Faculty: clinical teaching/research	6.1 (0.8)		6.2 (0.7)		6.0 (0.9)	
Full-time university Faculty: clinical focus	5.3 (1.3)		5.5 (0.7)		5.5 (1.0)	
Non-academic clinical practice	5.3 (1.1)		3.5 (1.3)		5.3 (1.0)	
Other/Undecided	5.3 (1.4)		5.5 (1.1)		5.1 (1.6)	

Abbreviations: CI, Confidence Interval, URiM, Underrepresented in Medicine (Black/African American or Hispanic/Latino); DO, Doctor of Osteopathic Medicine; MBBS, Bachelor of Medicine, Bachelor of Surgery.

Tests of significance were one-way analysis of variance on the measures.

^a^ Higher scores indicate stronger agreement with positive-training experiences at our institution.

^b^ Higher scores indicate stronger agreement with endorsement of academic-medicine career goals.

^c^ Higher scores indicate stronger agreement with positive-mentoring experiences at our institution among fellows who reported having a mentor and responded to the mentor role items.

^d^ One participant responded, ‘prefer not to answer,’ and with a single observation the analysis will not produce a standard deviation as there is no variability of data.

Fellows’ agreement with positive-training experiences, academic-medicine career goals, and positive-mentoring experiences also differed significantly by their career-activities intentions. Post-hoc tests (each *p* < 0.02) indicated that fellows with basic science teaching/research or clinical teaching/research faculty-career-activities intentions reported significantly greater agreement with positive-training experiences and academic-medicine career goals than fellows with non-academic clinical-practice intentions. Fellows with career-activities intentions as faculty with a clinical focus reported greater agreement with academic-medicine career goals than fellows with non-academic clinical-practice career-activities intentions (*p* *<* 0.001). Fellows with basic science teaching/research faculty-career-activities intentions reported greater agreement with positive-mentoring experiences than fellows with non-academic clinical practice intentions and with other/undecided career-activities intentions (each *p* < 0.05). No other significant associations were observed.

### Interviews

Twenty-four fellows across several clinical departments completed one-on-one interviews. Among interviewed fellows, three were offered and accepted a faculty position, three were offered and declined a faculty position, and 18 fellows were not offered a position. Fellows gave several reasons why they were not offered a position: they were too early in training; they had already decided to leave for family reasons or to accept an offer from another institution; our institution was not hiring; or they had decided to pursue private practice. Five themes emerged from analysis. Three themes described influences on decisions to accept a faculty appointment: career goals, lifestyle choices, and salary, benefits, and institutional resources. Two themes addressed communication about the institution’s priorities and potential faculty positions as well as workplace culture. Table 5 contains exemplar quotes for each theme.

**Table 5.** Exemplar quotes for each theme among 24 interviewed fellows (Denoting Each Fellow’s Self-reported Faculty-offer Group).

Theme 1: Fellows’ career goals *The sort of enticing thing is having some degree of flexibility to foster my own growth and help me along the pathway that I carve out for myself—and not necessarily one that is rigid, that somebody else carves out for me. (#4, offered/accepted)* *I did an MD to take care of patients. I mean, that’s probably the most important thing. (#6, offered/accepted)* *I don't want to do sort of your standard research that's easy to do here. I want to do research that's focused on medical education. And it didn't seem like there was a plan for that. (#13, offered/declined)* *I came in with a pretext of actually doing academic medicine and probably staying on as faculty at wherever I am. So, when I was actually interviewing for a fellowship programme, number one question I would ask is what is their retention rate of faculty members staying on... I wanted to do [a very specific aspect of my subspeciality]. They have said yes. I want to do some bench research. They've said yes. Next year I wanted to do some clinical educator, certification track, and they said I'm already going to be starting to do that next year. So everything's going as needed. (#1, not offered)*
Theme 2: Fellows’ lifestyle choices *I am not prioritising [compensation] as much. I know some places don't have control to, especially academic places … For me, my priorities are … more the schedule and the options for my husband and things like that, and location. (#17, not offered)* *I don't wanna do all of one thing. I wanna do a combination of all the things. When I first came here … I'm not from the area, but we had assumed that we would just stay for my fellowship and then move … where our family is. But right away, I felt really welcomed by the division, and pretty early on, I could see myself as staying on as faculty. (#12, not offered)* *I will say the overall kind of feel is more of ‘less life balance, more work,’ and, in order to get ahead, you must kind of do these kind of things—which, I think, does not come across well with fellows coming in as junior faculty, especially as there’s more of a push these days for work-life balance. (#7, not offered)*
Theme 3: Salary, benefits, and institutional resources *Basically, it comes down to sort of a value of education that I don't think WashU has… [P]romotions for medical education people are harder to come by here. (#13, offered/declined)* *Certainly there are a lot of people attracted that want to do more research. And this is a great place to do it because there's funding, there's access, there's infrastructure. I don't think that that's necessarily decreased. But I do think that there [is] an increase of people that want to do more clinical work. (#11, offered/declined)* *I think I have seen some people struggle—with their protected time to do research—because sometimes in the clinic, the patient’s right in front of you, so sometimes that can take over from the research component. But I think WashU, uh, especially our department, takes a strong stance in wanting to promote research. (#18, not offered)* *I think that my division chief offered me the most golden deal that she could … I mean, she was gonna protect one day a week for me to do some of those things [e.g., quality improvement projects] that I enjoy, which is a huge thing … you know, to have 20 percent of your time being focused on other activities besides clinical. I really can't … complain about what they were able to offer me as a full-time employee. (#11, offered/declined)* *It comes down to not only compensation but, again, making sure that I am able to have that continued education and supports. (#24, not offered)* *I just want to be a doctor and take care of patients, and then go home and be a mom. I felt like despite the fact that I was asking for a clinical position, I was gonna be asked to do a lot more than that, especially when I had already been told that I was gonna be 100 percent clinical-1.0 FTE. So, no protected time … I’d be working all the time taking additional call; I have no negotiating power with my salary; and was still being asked what research was I gonna be doing … It just felt like my goals no longer aligned with what the goals of the department and division were… (#10, not offered)* *It became kind of a, unfortunately, a no-brainer to not stay… I cried several days over it because it really hurt to turn down the offer 'cause I wanted to stay. But the offer that I was given … I was happy with 'cause it's certainly more than my fellow salary … but comparing it to the rest of the salaries, even in the region, that people were offering was shocking. But that's kind of a pervasive cultural aspect ... you hear quite frequently from the higher-ups that, well, it should be an honour to work here. And I think that there comes a time when that's not enough anymore. (#11, offered/declined)* *I think it’s kind of well-known that WashU does not pay as well as private practice in the area. But the monetary component is not as important to me as the work-life balance—and happiness with my department and with my group. It means more to me than an X amount of salary. (#18, not offered)* *So,… I took a job at … a smaller hospital, not a bigger one ... they offered me funding for … double what WashU offered me. That's a small thing, but that's a thing. The other institution also offered me six weeks of paternity leave—which WashU doesn't match that number … again, it was lots of little things, too, not just [the]compensation package. It was also lots of little pieces of, "Well, we'll also support your CME more." "Oh, we're gonna pay for your boards." "Oh, we're gonna pay for X, Y, Z, and Q," It's making my life easier, too. (#11, offered/declined)*
Theme 4: Communication about the institution’s priorities and potential faculty-position offers *I feel like all faculty, as soon as the fellow comes in, should just sit with … [the fellow] and … [ask], "Hey, what are your goals? What do you want to do?" … not tell you that you have to do research. What if that person doesn't want to do research and …wants to do private practice … I don't think you should force that person to do research. And if someone asks me…, "What research do you wanna do?" and I told them…, they should … [say], "Hey, you should go to that person right now. I'm gonna email them. Just go there and just talk to them..." So I think that … [faculty should] sit with a fellow and figure out their goal. (#1, not offered)* *I feel like all faculty, as soon as the fellow comes in, should just sit with … [the fellow] and my fellowship director really kept me in the loop the whole time about where they were... The bottom line is that they don’t have the clinical volume to support a 1.0 FTE … but she explained that she was motivated to try to keep me if she could and even went out of her way to look at figuring out,… [if] some other divisions within the department … might be able to use part of my time. I interviewed with some of those other divisions. I interviewed with the chair of the department … I really felt a sense of recruitment and that there was flexibility in the role. And then a little bit was just up to me. By this point though, I had already been told that there was not a clinical position, and I have started pursuing other options in other areas of the country. (#9, not offered)* *I’ve … started here as a resident … our … department chief was a different person … I think through a lot of leadership changes, those priorities [perception that medical education is a lower institutional priority] have begun to change … And I think that tone is … without question, set by leadership … I have seen the … overall culture of our division change substantially—um, after a new leader came in. And, um, I think the tone that leaders set is really important. (#4, offered/accepted)*
Theme 5: Workplace culture *But J1 waivers, they are hard for them to offer, so that would be the biggest hindrance. No matter how hard you want a fellow to stay on and how glorified … with his research, his CV, or anything, if he doesn't get that J1 waiver, he's not gonna stay. (#1, not offered)* *I have had very good experience in my collaborations and interactions with other departments. I have not found that to be the case within my own department… I think that the department that I am in is just not functioning in a way that is conducive to my career goals. (#16, offered/declined)* *I mean the culture here as a university is in my opinion phenomenal. It’s like very obviously high powered, and everyone knows it’s got like the name brand recognition and respect around the world ... it’s as elite as there exists. But at the same time, people here don’t carry themselves like that. So, there’s like a sense of humility and a very Midwesterner’s [way] that I like, you know. People are down to earth and they are easy to talk to. (#22, not offered)* *Actually, our division, they have a very difficult time … keeping people. And with high turnover rates, it's hard to motivate yourself to stay. (#11, offered/declined)* *Two female research-track young physicians who have raised concerns about mentorship within their labs, contracts have both been cancelled, or not renewed … after raising those concerns… [M]icroaggressions seem pretty common. On maternity leave, it’s really challenging to feel like you’re still on the same playing field as your male colleagues. (#3, not offered)*

### Theme 1: fellows’ career goals

Fellows’ goals and priorities both overlapped and diverged from the perceived institutional priorities for faculty. As a group, fellows perceived the institution’s priorities to include research, clinical care, and teaching—the tripartite mission of academic medicine—and they shared those priorities with the institution. Fellows emphasised the importance of a match between their career goals and a faculty-position offer, wishing for conversations about their career goals that could help frame an offer. However, fellows perceived department/division needs as determining job availability without adequate communication about whether positions would match a fellow’s goals and qualifications, leaving fellows wondering if a position might be offered.

Many fellows described academic medicine as a place to pursue their career goals. Once they decided to remain in academia, the key to fellows’ faculty-position decision-making at a particular institution was the match between their career goals and opportunities offered to them, including support for their career development, e.g., ‘*allowing me to do what I need to do clinically and continue to grow … giving me funding to complete my master’s.*’

### Theme 2: fellows’ lifestyle choices

Fellows’ career plans reflected their personal lifestyle choices. They described perceptions of living in the St. Louis area (which they considered family-friendly and affordable), its proximity to family, and whether professional opportunities for partners were available. ‘*We had to decide [if] St. Louis can be home for us, and we’ll figure out … how to stay close to [our extended family].’* Fellows’ lifestyle choices also affected their reactions to perceived happiness and work/life balance of the faculty, including expectations for working hours. While they did not expect potential offers to match their lifestyle choices, they observed the work/life balance that others maintained and decided whether this fits with their lifestyle.

### Theme 3: salary, benefits, and institutional resources

Salary and benefits offered by the institution played an important role in fellows’ decisions to stay or leave. Fellows recognised that salary may be lower in academia versus private practice; however, compared with other institutions, ‘*even in the region, [the salary] ... was shocking.’* For some fellows, however, salary was a lesser concern than opportunities to meet their career goals: ‘*I’d rather make … an academic salary and be happy than make a lot of money in a practice and feel lukewarm about my work.’*

Institutional resources that aligned with fellows’ career goals were incentives for considering a faculty position. Resources described included opportunities to engage in degree or certificate programmes, availability of outstanding research infrastructure (labs, equipment, collaborations, funding), availability of relevant mentors, and clinical resources (modalities of treatment, facilities, approaches to patient care, and presence of patients needing complex clinical care). Fellows commented, *‘The infrastructure for research that exists here doesn't really exist anyplace else,*’ and ‘*I like ... staying at a big academic institution that ... pulls in a lot of patients from all over the country and … world.’*

### Theme 4: communication about institutional priorities and potential faculty-position offers

Fellows discussed the need for communication between the fellow and their department/division faculty and fellowship directors. Fellows noted that being offered a faculty position depended primarily on department/division priorities and the tone set by institutional leadership. Whether or not a faculty offer was made relied heavily on the communication (or lack thereof) between the institution and fellows, including implicit or explicit communications about potential openings or a fellow’s needs and resources, such as start-up funds, to help a fellow achieve professional goals.

Communication with fellows in the early stage of their training helped address the department’s needs and foster fellows’ personal growth. ‘*We had very formative conversations … putting it all together and creating a role for me ... All of that culminated from a yearlong discussion…to the offer at the end, which was exactly what I was looking for, in terms of my own development and how I wanted to give back to the division.’*

Some fellows expressed disappointment with the communication. *‘I was told by our leadership that I would have an opportunity to … build a niche that was of … interest to me. Everyone in my division that I spoke to made it seem like my goals were exactly on par with what they wanted. But when I got the offer and spoke to the chair about it ... I was told that [my] goals were not in line with what the department needed, and the offer did not reflect the position that I had been [promised].*’ Fellows described how communication influenced whether or not they perceived they would be offered a faculty position or whether other job opportunities might be a better match with their career goals.

Fellows pointed out that leadership could address the lack of communication by being more transparent about the department’s/division’s needs. One fellow remarked, *‘if recruitment were just more open or transparent. Like if they could just say, ‘These are the gaps in our division or department, and this is where we need help in the next two or three years. This is what the responsibilities look like...’ It will help fellows make more informed decisions*.’

### Theme 5: workplace culture

The interpersonal environment shaped fellows’ perceptions of their workplace. *‘[Our University]'s a very well-known, well-respected institution, but it's still ... full of really nice, normal folks.*’ Fellows emphasised relationships with mentors and clinical and research team members as important to their decision about seeking a faculty position at our institution. Fellows who experienced positive relationships and support *‘look[ed] forward to coming to work … every day.*’ Others reported differently: ‘*There are certain attendings that are really intimidating ... They have a really good hold on the programme. And you can't argue."*

Other cultural concerns focused on faculty diversity, gender bias, and micro-aggressions. Some concerns were related to the clinical environment, illustrated by one fellow’s observation about ‘*how the nurses would treat female residents and especially female residents of colour … There’s a handful of people who really honestly tried to make some differences, but we didn’t see a whole lot of improvement.’* Attitudes in the workplace about work/life balance also contributed to fellows’ perceptions. One fellow commented, ‘*My priorities … changed to have a little better work-life balance. I think the opportunity for a more balanced position…is not something that's probable [here].*’

### Triangulation of survey and interview results

The interviews enriched our understanding of fellows’ decisions regarding accepting/declining a faculty position. In some instances, the surveys identified a mismatch between positions offered to fellows and fellows’ academic-medicine career goals; the interviews described the need for better communication about fellows’ career goals in relation to the institution’s needs and more detailed consideration of fellows’ individual career goals. Within the broad goal of working in an academic environment, many fellows described their specific goals to provide care for particular patient populations, pursue additional certificates or degrees (e.g., a master’s degree in education), or pursue a particular area of research. The surveys identified the importance of positive-training experiences and mentorship in fellows’ decisions to stay at the institution. In the interviews, the fellows articulated descriptions of workplace culture that influenced whether their experiences of training and mentorship were positive. As discussed below, the synthesis between survey and interview results informed ways in which an institution might intervene to shift workplace culture, communicate earlier and more fully, and consider the match between fellows’ individual career goals and the institution’s needs.

## Discussion

Eighty percent of survey respondents considered joining the faculty at WashU Medicine. This group reported stronger agreement with positive training and mentoring experiences and academic-medicine career goals than fellows who had not considered staying. Taken together, the survey and interview data identified factors associated with fellows' decisions to remain in academic medicine and accept a faculty position at our institution and described considerations important to their decisions, illuminating areas amenable to intervention to enhance faculty recruitment.

Many fellows who consider staying at their training institution will not be offered faculty positions, which may be limited by an institution’s or department’s capacity to hire new faculty or the alignment of fellows’ interests with departmental needs. The congruence between institutional/departmental needs and fellows’ career goals may vary by the institution’s mission, departments’ ability to hire new faculty, and the mix of faculty roles needed. For research-intensive institutions, the costs of research are consequential; in 2015, the AAMC reported that medical schools invested, on average, $0.53 per dollar of extramural funding received for research [27]. Institutional investment may be even higher in times when availability of extramural funding is threatened. Thus, it is important that research-intensive institutions hire clinicians to raise clinical revenue to help support the research and education missions [4]. Sixty percent of surveyed fellows expressed career-activities intentions as full-time university faculty in clinical teaching/research or other clinical foci; these parameters align with our institution’s expressed goal of expanding the clinical and clinical-research enterprise. However, 27 fellows who were offered a faculty position declined the offer, 13 of whom reported non-academic clinical practice or other/undecided career-activities intentions. It was surprising not only that these fellows were offered faculty positions that seemed misaligned with their career intentions, but also that 23 (41%) of 56 fellows who were not offered a position reported clinically relevant faculty career-activities intentions. It is imperative to better understand fellows’ career-activities intentions to promote retention of fellows as faculty, especially those with clinically relevant faculty interests. This is a prominent feature of *fellows’ career goals* in their faculty-offer decisions (Theme 1), emphasising the importance of discussing *fellows’ career goals* and seeking ways to match offers that meet the institution’s needs and fellows’ ambitions.

Single-specialty studies have reported varying proportions of fellows who remained as faculty after completing fellowship training, with reports of 30% [28] and 39% [29] of fellows in plastic surgery, 34% of fellows in paediatric emergency medicine [30], and 15% of fellows in breast surgical oncology [31]. When considering post-fellowship positions, other studies report that fellows consider compensation [32], work/life balance [10,32], family commitments [30], and how to balance competing demands of academia [33]. Our interviews support and further explain these earlier findings in fellows’ descriptions of balancing decisions with considerations of institutional *resources*, *salary, and benefits*, including protected time for research and/or teaching (Theme 3), and institutional *workplace culture* (Theme 5). Fellowship programmes should examine fellows’ career goals with consideration of their overarching concerns about work-life balance as a means to retain fellows whose professional priorities align with the needs and mission of the institution where they trained.

In addition to matching the needs of the institution with fellows’ career goals, both survey and interview data highlighted the importance of mentoring experiences and opportunities to fellows’ decision-making. The literature supports the importance of mentoring experiences to fellows’ decisions to accept (or decline) an offered position at their training institution [34,35]. Programme directors can work with fellows whose career goals align with an institution’s mission to identify mentors [13], which may benefit both fellows and the institution when fellows join the faculty by building upon the cooperative network of relationships formed during training [28]. Surveyed fellows with career-activities intentions in basic science teaching/research as well as those who graduated with dual MD/PhD degrees reported more positive training and mentoring experiences and endorsed academic-medicine career goals more than fellows who had other career-activities intentions (including fellows with clinical foci) and other degrees. Not surprisingly, many MD/PhD graduates aspire to academic-medicine careers [8,36,37], and many MD/PhD training programmes, especially those with federal MSTP funding, are expected to have clear programme objectives and processes to ensure that MD/PhD students are adequately mentored to succeed [38,39]. Further research at other institutions is needed to compare and contrast the findings we saw at our institution, a research-intensive medical school with one of the oldest and largest MSTPs in the U.S. Conducting our study with fellows training at other academic institutions and understanding the reasons that fellows training at other research-intensive and non-research-intensive medical schools may, or may not, be offered a faculty position (and fellows’ reasons for accepting or not accepting these positions) are worthy areas for additional research, as the results from other schools may be different from those reported by fellows at our institution. Additionally, programme directors should place greater emphasis on mentoring programmes tailored to physicians with clinically-focused academic interests if clinical-care delivery is a major focus of the institution. Our study highlights the potential need for mentoring of fellows pursuing clinical teaching/research and other clinically focused faculty careers in research-intensive institutions. Connecting with fellows early in training to gauge their career-activities intentions and match them with faculty mentors with similar career interests may facilitate negotiating a viable faculty position, satisfying the needs of fellows and the institution.

Also amenable to intervention are *workplace culture* (Theme 5) and *clarity of communication about potential positions* (Theme 4), both of which played important roles in fellows’ decisions. Several interviewed fellows described ‘bait and switch’ experiences, where offered faculty positions did not include either previously discussed protected time for research or clinical service hours aligned with their professional interests. Communication about fellows’ priorities and whether they are aligned with perceptions of institutional priorities remains key. To intervene, our institution adjusted our culture that over-emphasised research-focused careers while needing clinicians, in part by developing dedicated clinical tracks in fellowships. To improve communication about potential positions, our institution provided explicit instructions to programme directors, divisions, and departments to begin the communication and recruitment process earlier and be more inclusive of clinicians. Providing leaders with guidance about clear communication about whether fellows may be offered a faculty position addresses a concern expressed by interviewed fellows and helps inform decision-making. Identifying and communicating the institution’s priorities for faculty positions helps fellows discern whether their career goals match the needs of the institution. Communication with fellows about their career goals helps leaders to consider whether offers can be individualised to support fellows’ goals while also addressing the needs of the institution. Attending to other aspects of workplace culture is a more complex task, although worthy of attention. Many aspects of workplace culture, including experiences of bias, inclusion, diversity, relationships, and fellows’ perceptions of the possibility of work/life balance influence job satisfaction, desire to remain in academic medicine, and desire to remain at an institution. This mixed-methods study describes a process that other institutions can use to discern institutional factors that influence fellows’ decisions about whether to remain at the institution where they completed their fellowship training. The survey data can provide an overview of fellows’ training and mentoring experiences and interests in a career in academic medicine. Additionally, key themes identified in the qualitative analysis of the interview data, particularly about the need for clarity and consistency in communicating with fellows about possible faculty positions, seeking opportunities to ask about and discuss fellows’ career goals, and addressing aspects of institutional culture to create a supportive work environment, can provide guidance for other institutions striving to maximise retention of fellows who are interested in pursuing faculty positions at their institution.

This study has several limitations. First, we report survey and interview data from a single, research-intensive institution, and survey participation was low. Other studies have reported low response rates from physicians and trainees [40–43] and lower average response rates from physicians compared with non-physicians surveyed (54% vs. 68%, respectively) [40]. Other studies report even lower response rates between 27% and 46%, varying between trainees and physicians in practice and among different specialties [42,43]. While low response rates increase risk of response bias, as an exploratory study, our intention was to generate hypotheses for further research and identify where we might intervene at our school. The generalisability of our findings to other institutions with different missions or research, educational, and/or clinical opportunities may also be limited. Factors associated with fellows’ decisions to train at our research-intensive institution may be similar to or different from their decisions to accept offered faculty positions, and there may be other variables that fellows consider in their decisions but were not identified here. We are unaware of specific selection criteria when considering to offer a faculty position to fellows in training, and several interviewed fellows described frustration by the lack of clear and consistent communication regarding faculty-selection criteria. Hiring new faculty is limited by a department’s needs [44], and while we are unaware of any specific criteria used in faculty-offer decisions (e.g., availability of positions and the quality and/or quantity of a fellow’s scholarly work), our study highlights an opportunity for fellowship programmes to engage in clear and consistent communications with fellows who may be considering or hoping to join the faculty at the institution where they train. Our qualitative findings about communication pertain to all fellows, whether they want to pursue research or more clinically focused careers, suggesting that communication between each fellow and their division chief/department chair is essential, and important regardless of the institution where the fellow trains. Finally, our anonymous, cross-sectional, sequential triangulation mixed-methods study precluded us from linking survey and interview data for fellows who participated in both parts of the study, examining differences between fellows who did and did not participate in both the survey and interview, and following up with fellows who were too early in training to have been offered a faculty position. Moreover, we cannot make causal inferences from the associations we observed.

## Conclusions

To our knowledge, this is the first mixed-methods study examining fellows’ decision-making to accept or decline faculty positions where fellowship training was completed. As such, our study contributes new knowledge about attitudinal and experiential factors that fellows may consider in career decisions, fellows’ reasons for accepting or not accepting offered faculty positions, and what institutions can do to encourage fellows to join their faculty after training. Our study identifies actionable opportunities for other fellowship programmes to think about when considering fellows for faculty positions in the context of department needs. For example, attending to fellows’ career goals, promoting positive training and mentoring experiences, and proactively and intentionally communicating about potential faculty positions can foster informed decisions about joining the faculty. Understanding fellows’ reasons for accepting or declining a faculty position at the institution where they trained should help fellowship programmes train and support fellows to achieve their career goals and better enable institutions to retain faculty candidates whose goals align with the institution’s needs and priorities.

## Supplementary Material

Supplementary Material Survey Items.docx Supplementary Material Survey Items.docx

Supplementary Material Interview Guide.docx Supplementary Material Interview Guide.docx

## Data Availability

It is not possible to share research data publicly as individual privacy could be compromised.
