# Supplementary material for: Retention of fellows to the faculty at a research-intensive academic medical center: a mixed-methods study
Source: Med Educ Online. 2026 Aug 7;31(1):2714642. doi: 10.1080/10872981.2026.2714642 (PMC13455691; doi:10.1080/10872981.2026.2714642)
Supplement: Supplementary file 1 [file ZMEO_A_2714642_SM5087.docx]

Survey Items

[Institution Name]’s Mission:

"[Institution Name] will lead in advancing human health through the best clinical care, innovative research and the education of tomorrow's leaders in biomedicine in a culture that supports diversity, inclusion, critical thinking and creativity."

For each of the following statements, please indicate your level of agreement.

| Item | Strongly Disagree | Disagree | Slightly Disagree | Neither Agree nor Disagree | Slightly Agree | Agree | Strongly Agree |
| --- | --- | --- | --- | --- | --- | --- | --- |
| My professional values are well aligned with [this institution’s] mission |  |  |  |  |  |  |  |
| I am satisfied with the role my training program has had in my career advancement. |  |  |  |  |  |  |  |
| [This institution] provides a collegial and inclusive work environment for me. |  |  |  |  |  |  |  |
| [This institution] provides a supportive work environment for me. |  |  |  |  |  |  |  |
| I am confident in my ability to progress in my career path.^a^ |  |  |  |  |  |  |  |
| [This institution] is committed to my career success. |  |  |  |  |  |  |  |
| [This institution] facilitates my professional development. |  |  |  |  |  |  |  |
| I can see myself living in [city name] or surrounding communities over the long term.^a^ |  |  |  |  |  |  |  |
| I am interested in pursuing a career in academic medicine. |  |  |  |  |  |  |  |
| A competitive salary is an important factor to me as I consider a position after completing fellowship.^a^ |  |  |  |  |  |  |  |
| Being able to practice clinically in my area of specialty training is an important consideration as I plan my professional career path.^a^ |  |  |  |  |  |  |  |
| Protected time to conduct research is an important consideration as I plan my professional career path. |  |  |  |  |  |  |  |
| Participation in education of others is an important consideration as I plan my professional career path. |  |  |  |  |  |  |  |
| I am satisfied with the mentoring I have received during fellowship. |  |  |  |  |  |  |  |
| I am satisfied with the career advice I have received during fellowship. |  |  |  |  |  |  |  |
| My fellowship training has provided me with opportunities to gain expertise in non-clinical career areas of interest to me. |  |  |  |  |  |  |  |

^a^Four items were sequentially dropped from further analysis after four iterations of the principal components analysis (PCA) as these items were not descriptive of either of the two new variables (i.e., components) that emerged from the PCA.

Do you have a mentor at [institution name]?

a. Yes
b. No

(Only for those who answered “yes” to having a mentor) For each of the following statements, please indicate your level of agreement.

| Item^a^ | Strongly Disagree | Disagree | Slightly Disagree | Neither Agree nor Disagree | Slightly Agree | Agree | Strongly Agree |
| --- | --- | --- | --- | --- | --- | --- | --- |
| My mentor thinks highly of me. |  |  |  |  |  |  |  |
| My mentor guides my professional development. |  |  |  |  |  |  |  |
| My mentor suggests strategies for achieving career aspirations. |  |  |  |  |  |  |  |
| My mentor brings my accomplishments to the attention of important people. |  |  |  |  |  |  |  |
| My mentor has encouraged me to pursue a position here at [institution name]. |  |  |  |  |  |  |  |

^a^The first four items were previously published [1, 2].

At this point in your fellowship, please indicate your career intention from the activities below:

a. Full-time University Faculty: Basic science teaching/research
b. Full-time University Faculty: Clinical teaching/research
c. Full-time University Faculty: Clinical Focus (including quality and safety or clinical practice/leadership)
d. Non-academic Clinical Practice
e. Non-academic Research Scientist
f. Other (please specify in box below)
g. Undecided

Over the course of your fellowship training, have you considered staying at [institution name] as a member of the faculty?

a. Yes
b. No

Have you been offered a faculty position at [institution name] after you complete fellowship training?

a. Yes
b. No
c. Too early

(Only for those who answered “yes” to “Have you been offered a faculty position at [institution name] after you complete fellowship training?”) Did you accept the offer of a faculty position at [institution name]?

a. Yes
b. No

Please answer the following demographic questions.

With which gender do you identify?

a. Female
b. Male
c. Non-binary
d. Prefer to self-describe (please use box below) _____________
e. Prefer not to answer

With which group(s) do you identify (select as many that apply)?

a. American Indian or Alaska Native
b. Asian
c. Black/African American
d. Native Hawaiian or Other Pacific Islander
e. White
f. Prefer not to answer

Are you of Hispanic/Latinx/Spanish origin?

a. Yes
b. No
c. Prefer not to answer

What is your marital status?

a. Married
b. In an unmarried partnership
c. Divorced
d. Separated
e. Widowed
f. Single, never been married
g. Prefer not to answer

What is your year of fellowship training?

a. Year 1
b. Year 2
c. Year 3
d. Year 4 or more

Department (please select from the drop-down list)

a. Anesthesiology
b. Emergency Medicine
c. Medicine
d. Neurology
e. Neurosurgery
f. Obstetrics and Gynecology
g. Ophthalmology and Visual Sciences
h. Orthopaedic Surgery
i. Otolaryngology
j. Pathology and Immunology
k. Pediatrics
l. Psychiatry
m. Radiation Oncology
n. Radiology
o. Surgery
p. Prefer not to answer
2. What is your terminal degree or degrees?
   a. MD
   b. DO
   c. MD/PhD
   d. Other (please specify in box below) ___________
3. Please indicate the amount of total debt (from all sources) you currently owe.
   a. No debt ($0)
   b. $1 to $49,999
   c. $50,000 to $99,999
   d. $100,000 to $149,999
   e. $150,000 to $199,999
   f. $200,000 to $249,999
   g. $250,000 to $299,999
   h. $300,000 or more
   i. Prefer not to answer
4. To what degree has your level of debt influenced your career plans?
   a. No influence
   b. Minor influence
   c. Moderate influence
   d. Strong influence

References

1.	Ragins BR, McFarlin DB: Perceptions of mentor roles in cross-gender mentoring relationships. *Journal of Vocational Behavior* 1990, 37(3):321-339.

2.	Dilmore TC, Rubio DM, Cohen E, Seltzer D, Switzer GE, Bryce C, Primack B, Fine MJ, Kapoor WN: Psychometric Properties of the Mentor Role Instrument When Used in an Academic Medicine Setting. *Clinical and Translational Science* 2010, 3(3):104-108.
