# Supplementary material for: Retention of fellows to the faculty at a research-intensive academic medical center: a mixed-methods study
Source: Med Educ Online. 2026 Aug 7;31(1):2714642. doi: 10.1080/10872981.2026.2714642 (PMC13455691; doi:10.1080/10872981.2026.2714642)
Supplement: Supplementary file 2 [file ZMEO_A_2714642_SM5088.docx]

**Semi-Structured Interview Guide**

Thank you for agreeing to be interviewed for this study. We want to learn more about fellows’ experiences here and their interests in staying at [our institution name] after fellowship as a member of the faculty.

1. During your fellowship, have you considered pursuing a faculty position here after fellowship? Why or why not?
2. Have you initiated or engaged in conversations with your division chief or department chair about pursuing a faculty position here to discuss your professional goals with them?
   a. Why or why not?
   b. If they engaged in conversations with leadership:
      i. How did those meetings go with regard to your career plans?
      ii. What did they tell you that made you think that your career goals were a good fit (or not a good fit) with the department’s or division’s needs?
3. What do you think are the most important factors that your Division and Department heads consider when thinking about appointing a fellow in your program to the faculty?
4. Have you been offered a faculty position at [our institution name] after you complete fellowship training?
   a. If Yes (they have been offered a faculty position at [our institution name]), ask: Did you accept the position?
      i. If yes (they accepted the faculty position), ask:
         a) To what rank and track will you be appointed? (Rank: Instructor or Assistant Professor; Track: Investigator, Clinician, or Research)?
         b) What were your reasons for accepting a faculty position here at [our institution name]? Probes, if not mentioned spontaneously:
1) Location near family?
2) Offered desired balance of protected time to pursue research/education/other scholarly interests?
3) Want to spend more time in clinical practice or in a specific type of clinical practice?
4) Amount of money offered?
5) Your career goals meshed with [our institution name] or with the Department’s needs
6) Comfort with the culture at [our institution name]?
7) Concerned about life/lifestyle or ability to build life you want in [city name] area?
8) Spouse/partner needs and job opportunities?
9) Other reasons.
   ii. If No (they did not accept the faculty position), ask:
       a) What were your reasons for not accepting a faculty position here at [our institution name]? Probes, if not mentioned spontaneously:

1) Location near family?

2) Not offered desired balance of protected time to pursue research/education/other scholarly interests?

3) Want to spend more time in clinical practice or in a specific type of clinical practice?

4) Amount of money offered?

5) Your career goals did not mesh with [our institution name] or with the Department’s needs?

6) Bad experience here?

7) Discomfort with the culture at [our institution name]?

8) Concerned about life/lifestyle or ability to build life you want in [city name] area?

9) Spouse/partner needs?

10) Other job opportunities?

11) Other reasons?

b. If No (they were not offered a position):

i. (If they wish they had), ask: Why do you think you were not offered a faculty position? Probes:
   a) Not a good fit?
   b) Mismatch between desired balance of protected time to pursue research/other scholarly interests and needs of division/department?
   c) Your career goals did not mesh with [our institution name] or with the Department’s needs?
   d) Interpersonal conflicts?
   e) Other reasons?
ii. (If they did not want to stay at [our institution name] anyway), ask: What are some of the reasons that you did not want to stay at [our institution name] on the faculty after fellowship training? Probes:
    a) Location near family?
    b) Not a good fit?
    c) Mismatch between desired balance of protected time to pursue research/other scholarly interests and needs of division/department?
    d) Your career goals did not mesh with [our institution name] or with the Department’s needs? Bad experience here?
    e) Interpersonal conflicts?
    f) Spouse’/partner’s needs?
    g) Other job opportunities?
    h) Other reasons?
iii. (If it was too early in fellowship), ask: What are some of the reasons you might want to stay on the faculty at [our institution name] after fellowship? Probes:
     a) Location?
     b) Desired balance of protected time to pursue research/education/other scholarly interests?
     c) Want to spend more time in clinical practice or in a specific type of clinical practice?
     d) Your career goals mesh with [our institution name] or with the Department’s needs?
     e) Comfort with the culture at [our institution name]? Will be able to have the life/lifestyle I want to live?
     f) Ability to build life you want in [city’s name] area?
     g) Spouse/partner needs and job opportunities?
     h) Other reasons
iv. (If it was too early in fellowship), ask: Are there any reasons why you might not consider a faculty position at [our institution name] after you complete fellowship training? Probes:
a) Location?
b) Not a good fit?
c) Mismatch between desired balance of protected time to pursue research/other scholarly interests and needs of division/department?
d) Your career goals do not mesh with [our institution name] or with the Department’s needs?
e) Bad experience here?
f) Interpersonal conflicts?
g) Spouse’/partner’s needs?
h) Other job opportunities?
i) Other reasons?
5. How much value do you think [our institution name] places on each of the following faculty roles at [our institution name]? Please rank from highest (1) to lowest priority (4):

Clinical practice

Teaching/education

Research in clinical and/or basic sciences

Medical education research/scholarship

6. And if you had to rank your priorities, what would yours be?
